# Use of Artificial Intelligence in Preoperative Planning in Surgery: A Narrative Review

**DOI:** 10.7759/cureus.102187

**Published:** 2026-01-24

**Authors:** Sood Alsairefi, Retaj Alawadhi, Sara Abdulaziz, Rawan Al Hashemi, Nagi Alqallaf, Jana Mohammad, Butti Albutti

**Affiliations:** 1 Department of Plastic and Reconstructive Surgery, Jaber Al-Ahmad Hospital, Ministry of Health, Kuwait City, KWT; 2 Department of Otolaryngology-Head and Neck Surgery, Jaber Al-Ahmad Hospital, Ministry of Health, Kuwait City, KWT; 3 Department of General Surgery, Tawam Hospital, Sheikh Tahnoon Bin Mohammed Medical City, Al Ain, ARE; 4 Department of General Surgery, Jaber Al-Ahmad Hospital, Ministry of Health, Kuwait City, KWT

**Keywords:** ai and machine learning, artificial intelligence, deep learning artificial intelligence, machine learning (ml), plastic and reconstructive surgery, plastic surgery, preoperative planning, pre-surgical planning, surgery general, virtual surgical planning (vsp)

## Abstract

The integration of artificial intelligence (AI) into preoperative planning for surgery has shown significant potential for enhancing surgical outcomes. AI technologies, such as machine learning (ML), deep learning, three-dimensional (3D) modeling, and predictive analytics, are increasingly being used to improve the accuracy of surgical planning, risk stratification, and patient outcomes.

The aim of this narrative review was primarily to evaluate the applications, effectiveness, and limitations of AI in preoperative surgical planning. This review synthesized findings from recent studies published between 2020 and 2025, focusing on the use of AI in preoperative planning across surgical specialties, with particular attention to well-documented examples from plastic and reconstructive surgery.

AI technologies, including ML algorithms, 3D modeling, and predictive analytics, have shown promise in potentially improving surgical precision, reducing complications, and enhancing patient satisfaction across multiple surgical procedures. However, challenges related to data heterogeneity, publication bias, and the limited number of large-scale prospective multicenter clinical studies remain significant barriers to its broader implementation.

AI can potentially support preoperative planning in surgery by augmenting surgical decision-making and contributing to improved clinical outcomes. Integrating AI with emerging technologies, such as 3D printing and virtual reality, could further expand its applications in surgical planning and patient care.

## Introduction and background

The integration of artificial intelligence (AI) into healthcare is one of the most transformative developments in modern medicine, with surgical specialties being the most significantly impacted by this technological revolution. AI-related developments are now widely discussed in the scientific community, with considerations of the theoretical aspects and practical applications in various areas of society [1]. AI refers to computing systems that perform skills normally requiring human intelligence, such as problem-solving, visual perception, and reasoning [2]. In addition, machine learning (ML) constitutes a specific set of techniques within AI, predicated on processes ranging from learning to modeling patterns in data [2]. The mathematical foundation of ML predominantly depends on traditional statistics, hence, the term "statistical learning," with algorithms proving particularly useful in medicine for predicting specific outcomes [2].

Recent advances in AI have demonstrated particularly promising applications in various surgical fields in which precise preoperative planning is critical for optimal outcomes. Examples include breast reconstruction, where AI technologies have supported the selection and planning of deep inferior epigastric perforator (DIEP) flap procedures via enhanced computed tomography (CT) angiography analysis [3,4]. AI algorithms have specifically been developed to predict the vascular anatomy in the abdominal region and optimize perforator selection, with semi-automatic identification methods matching manual accuracy for larger vessels while reducing the preoperative analysis time by two hours per patient [4]. Deep learning algorithms using the U-Net architecture advance automatic segmentation capabilities, thereby reducing clinician labor and variability in image analyses while supporting efficient tumor size and volume measurements [4]. In other areas, such as facial surgery, ML models have demonstrated potential capabilities in rhinoplasty planning, whereby convolutional neural networks (CNNs) are able to recognize hidden patterns and predict outcomes with an accuracy that surpasses human capabilities [5]. A ranking CNN algorithm outperforms human references in age estimation, with a correlation coefficient of 0.9, whereas three-dimensional (3D) image registration technology enables the accurate estimation of forehead flap dimensions for nasal defect reconstruction [5]. These plastic surgery-specific applications exemplify the transformative potential of AI in preoperative planning, in which ML algorithms can process vast databases of perioperative photographs to produce realistic simulations and enhance surgical decision-making for reconstructive and aesthetic procedures. These examples from plastic and reconstructive surgery illustrate the possible role of AI in preoperative planning, while similar approaches are being explored across general, cardiothoracic, orthopedic, and other surgical disciplines.

AI has been rapidly integrated into all aspects of human life, with continuous research being conducted on its applicability in medicine and surgery [6]. This application focuses on screening and interventions to improve the quality and safety of patient care [7]. ML, as a subset of AI, has gained tremendous popularity in healthcare, especially in surgery, and it involves algorithms and input data that produce the expected outputs [7,8]. The global AI healthcare market has remarkable growth potential, with a compound annual growth rate of 43.4% expected from 2022 to 2030, increasing from $7.9 billion in 2021 to an estimated $201.3 billion by 2030 [7].

AI has emerged as a transformative technology in preoperative surgical planning, with applications in risk prediction, 3D modeling, and imaging analysis [9]. AI algorithms can analyze a patient's data to predict individual risk profiles and identify optimal surgical candidates by integrating medical histories, laboratory values, and imaging studies [9]. AI algorithms are used preoperatively to analyze medical records and images, offering personalized treatment plans and risk assessments to optimize planning [10]. In plastic surgery, the use of AI applications has increased, with innovative approaches emerging across the surgical continuum from preoperative planning to postoperative evaluation [11]. AI-powered simulations involve advanced algorithms to produce visual representations of potential surgical outcomes during preoperative consultations [12]. The first widespread use of AI was in the computer augmentation of human performance, with clinician-machine interaction demonstrated to augment decision-making [13]. Technologies such as image-guided surgery systems improve surgical orientation and patient outcomes by helping surgeons identify anatomical structures quickly and reliably [14]. AI and robotics have revolutionized surgical practices by enabling minimally invasive procedures using robotic systems that provide precise tissue assessment feedback [15]. In orthopedics, AI applications have increased with an emphasis on improving diagnostic accuracy, treatment planning, and patient care [16,17]. Advanced imaging techniques generate vast amounts of data that AI algorithms, particularly deep learning models, can use to automate anatomical structure segmentation [18]. The emergence of 3D CNNs represents a significant evolution in deep learning applications and provides a multidimensional analysis that surpasses traditional diagnostic methods [19]. Large language models, such as ChatGPT (OpenAI, San Francisco, CA, USA), show another AI application approach, with ML-based chatbots improving communication performance by learning from vast training data [20].

Despite significant technological progress, several fundamental barriers hinder the successful integration of ML into clinical surgical practice. Research efforts are often limited to isolated institutional studies, with fewer than 40% adhering to algorithm-specific reporting standards such as the Transparent Reporting of a multivariable prediction model for Individual Prognosis Or Diagnosis (TRIPOD) + AI (TRIPOD-AI) [11]. The complexity of AI systems makes interpretation challenging in surgical applications and requires careful integration with surgeon expertise and clinical judgment [21]. In addition, data integrity is crucial because AI learning quality depends on data input quality, and verification mechanisms have significant limitations that undermine AI system reliability [21]. Ethical concerns, such as privacy, trust, consent, and conflicts of interest, also present challenges, with patient confidentiality at risk, owing to the extensive datasets required for ML [22]. The General Data Protection Regulation emphasizes the need for clear patient consent, whereas a lack of professional training and algorithmic transparency further hinders healthcare providers' confidence in ML systems [22]. Finally, ethical implications, including algorithmic biases across diverse demographics and emotional dynamics overlooked by robotic process automation, exacerbate these challenges [11,15,22].

Rationale

With the rapid evolution of technology, the transformative potential of AI in surgery, combined with existing implementation challenges, necessitates a comprehensive evaluation of current applications, limitations, and future directions. AI technology represents a transformative era, characterized by enhanced precision, personalized care, and improved surgical outcomes with advanced data analytics and ML capabilities that facilitate complex image processing, real-time decision-making, and predictive modeling [21]. This technology is a valuable tool for preoperative planning, intraoperative guidance, and postoperative care, with the potential to elevate surgical practice standards [21]. However, successful AI implementation depends on seamless integration into existing clinical workflows, which require user-friendly interfaces, interoperability with electronic health records, and minimal additional time for adoption in busy surgical practices [9].

The rapid increase in AI-related publications post-2020 highlights growing interest in AI and its potential in surgical practice. However, focused reviews examining the role of AI in the preoperative planning of surgery are notably lacking. This gap in the literature presents a critical opportunity to examine the specific applications and limitations of AI in this domain. Addressing these gaps will provide valuable insights for overcoming implementation challenges and identifying strategies for enhancing the practical integration of AI into surgical workflows.

An updated, focused review is essential to consolidate the evidence on preoperative planning in surgery, which will inform clinical practice, identify barriers to adoption, propose future directions, and ultimately support more efficient and patient-centered care. As AI continues to be implemented, its applications in surgery show significant promise for improving outcomes and patient safety, with surgeons leveraging AI to swiftly interpret complex data and pave the way for innovative solutions to reduce medical problems [21]. Therefore, the aim of this comprehensive analysis was to address the gap between AI technological capabilities and practical surgical implementation within a balanced framework that upholds the highest patient care standards while fostering innovation and ensuring ethical AI use in the surgical field [21].

Objectives

The primary objective was to evaluate the applications, effectiveness, and limitations of AI in the preoperative planning of surgical procedures. The secondary objectives were as follows: (1) to identify specific AI technologies, including ML algorithms, 3D modeling, predictive analytics, and other emerging technologies, used in the preoperative phases; (2) to assess reported outcomes, such as potential improvements in surgical precision, reduced complications, and enhanced patient satisfaction; (3) to explore challenges, including ethical issues, data biases, and implementation barriers; and (4) to highlight opportunities for future research and integration with emerging technologies such as 3D printing and virtual reality (VR).

## Review

Methods

Study Design and Protocol Registration

The aim of this narrative review was to explore the application of AI in preoperative planning for surgery by providing a broad descriptive synthesis of the literature on the use of ML, deep learning, natural language processing, and computer vision. This review did not follow the Preferred Reporting Items for Systematic Reviews and Meta-Analyses (PRISMA) 2020 guidelines or prospectively register a protocol. It instead summarizes the current state of AI applications across various surgical procedures.

Eligibility Criteria

This review included English-language studies published between 2020 and 2025 that addressed AI applications in preoperative surgical planning. Studies were included if they focused on risk stratification, imaging analysis, surgical simulation, or patient education in surgical contexts. A broad range of surgical procedures was considered. Studies were excluded if they focused primarily on intraoperative or postoperative phases, were non-clinical, or were editorials/conference abstracts without full text.

Search Strategy

A comprehensive literature search was conducted across the following databases: PubMed/MEDLINE (US National Library of Medicine, Bethesda, MD, USA), Scopus (Elsevier, Amsterdam, Netherlands), Web of Science (Clarivate Analytics, Philadelphia, PA, USA), Embase (Elsevier, Amsterdam, Netherlands), Cochrane Library (The Cochrane Collaboration, London, UK; published by Wiley, Hoboken, NJ, USA), and Google Scholar (Google LLC, Mountain View, CA, USA). The search terms included combinations of "artificial intelligence", "machine learning", "deep learning", "neural network", "preoperative", "surgical planning", and "plastic surgery". The search strategy was adapted for each database, and the reference lists of relevant studies were manually searched. A forward citation search for crucial studies was conducted by using Google Scholar.

Study Selection Process

Studies were selected based on thematic relevance to preoperative AI applications in surgery. Data were synthesized descriptively to capture broad trends, focusing on AI methodology, surgical context, and reported outcomes. No formal quantitative meta-analysis was performed.

Data Collection Process

A general thematic approach was used to collect the relevant information. The main aspects included the type of preoperative application (e.g., risk stratification, imaging, and surgical simulation) and outcomes (e.g., accuracy, sensitivity, specificity, clinical impact, and patient outcomes). A detailed, structured extraction form was not used. The data were instead synthesized to capture broad trends and insights, focusing on the integration of AI in clinical workflows and its impact on preoperative planning.

Data Synthesis and Analysis

This narrative review provides an overview of AI applications in preoperative surgery and highlights trends, successes, and challenges. Quantitative analysis was not conducted; thus, the synthesis emphasized a descriptive understanding of the use of AI in this field, highlighting patterns and emerging themes across the included studies.

Results

AI for Risk Stratification

Multiple AI technologies have been applied for preoperative risk assessment across surgical specialties, demonstrating superior performance compared with traditional risk assessment tools [23,24]. The integration of AI algorithms in surgical practice has revolutionized preoperative planning by enabling the precise analysis of patient data to identify ideal candidates for specific procedures and to predict individual risk profiles through diverse inputs, including medical history, laboratory values, imaging studies, and social determinants of health [6]. ML algorithms, such as gradient boosting, random forest, support vector machines, and neural networks, have demonstrated performance higher performance than that of traditional risk assessment tools in various settings [24].

For example, in cardiac surgery, ML models significantly outperform the European System for Cardiac Operative Risk Evaluation (EuroSCORE) II risk model and logistic regression models in predicting mortality after elective procedures [21]. The eXtreme Gradient Boosting (XGBoost) algorithm achieved the highest predictive accuracy for acute kidney injury, with an internet-based estimator providing real-time risk assessment [24]. The patient tracking list tool implemented in the National Health Service hospitals achieved area under the receiver operating characteristic curve (AUROC) values of 0.95 (95% confidence interval (CI): 0.92-0.98) for mortality prediction and 0.80 (95% CI: 0.78-0.82) for postoperative complications [25]. A random forest model for risk of intensive care unit admission revealed a sensitivity of 73.3% and specificity of 80.8% under prospective conditions [25].

Advanced AI applications have extended beyond traditional risk assessments to encompass comprehensive preoperative planning, utilizing algorithms that analyze medical records and imaging to offer personalized treatment plans and risk assessments [7]. A gradient-boosted decision-tree algorithm, using data from 1,477,561 patients, predicted 30-day mortality and major adverse cardiac and cerebrovascular events with high sensitivity and specificity [26]. Random forest models achieved an accuracy of 84.7% and an AUROC of 87.3% with feature importance analysis, identifying body mass index, age, preoperative comorbidities, and surgical approach as significant contributors to complications prediction [27,28].

ML models demonstrated strong performance in predicting complications (AUROC: 0.84), pain (AUROC: 0.83), and patient-reported outcomes (AUROC: 0.81), but were less accurate for readmission/reoperation (AUROC: 0.66) [28]. An ML model using intraoperative nociception data predicted moderate-to-severe postoperative pain with a cross-validated AUROC of 0.753 [28]. AI algorithms can individualize surgical recommendations, using preoperative data, particularly in complex decision-making scenarios such as bariatric surgery selection [28,29]. Risk prediction models constitute 51% of the AI applications in surgery, with 39% focusing on risk assessment improvement [23].

AI for 3D Simulation

AI-enhanced 3D modeling technologies have demonstrated potential improvements in preoperative surgical planning across multiple specialties, with particular advancements in surgical visualization and patient-specific modeling [6]. The integration of advanced imaging analysis and 3D modeling has been revolutionized by AI with deep learning algorithms that automatically segment anatomical structures in CT or magnetic resonance imaging (MRI) scans [6]. AI algorithms for image interpretation and processors dedicated to AI functions significantly reduce the technical and financial requirements for obtaining detailed three-dimensional images of a patient's anatomy [30].

In plastic surgery applications, computer vision and deep learning algorithms analyze preoperative images to generate realistic visualizations of the expected surgical outcomes, thereby helping align patient expectations with achievable results [6]. AI-powered simulations in cosmetic surgery involve advanced algorithms that produce visual representations of potential surgical outcomes during preoperative consultations and facilitate communication between patients and surgeons [9]. An AI-assisted surgical simulation system, LungDimensionGo V1.0 (SunKun, Beijing, China), adopted the EfficientDet method (Google Research, Brain Team, Mountain View, CA, USA) to detect lung nodules and used an image segmentation algorithm, based on deep learning models Mamba-UNet (Ziyang Wang, Jian-Qing Zheng, Yichi Zhang, Ge Cui, and Lei Li (Cornell University, Ithaca, NY, USA)) and SegRefiner (Mengyu Wang, Henghui Ding, Jun Hao Liew, Jiajun Liu, Yao Zhao, and Yunchao Wei (Institute of Information Science, Beijing Jiaotong University, Beijing, China)), to reconstruct 3D models of the lungs by using CT images [31]. Segmentations of skeletal and visceral structures were obtained using the TotalSegmentator extension (University of Freiburg, Freiburg, Germany), with an average processing time of <5 minutes [30].

VR-assisted planning utilizes VR technology to enable precise surgical preparation using patient-specific models and is often combined with augmented reality (AR) technology during surgery by displaying additional anatomical information [11]. Compared with traditional methods, the AI-assisted methods have shorter model reconstruction times, higher accuracy of anatomical structures, reduced operative times, less intraoperative blood loss, shorter postoperative chest tube duration, reduced postoperative hospital stays, shorter total hospital stays, and fewer postoperative complications [31].

AI has been applied to address the largest barrier to implementing 3D printing by automating manual processes through algorithms that efficiently process the large amounts of data required to prefabricate models, convert them through a slicer, and detect and rectify errors [32]. CNNs enable 3D-printed organ models in preprocessing to improve the accuracy and reliability of medical images, whereas Open-Loop AI printing makes printing on complex surfaces feasible by using predictive models combined with ML [33,34]. In orthopedics, AI applications in total hip arthroplasty preoperative planning include image segmentation, 3D reconstruction, implant selection, and virtual surgery simulations [13,14].

VR applications integrated with AI demonstrated clinical utility, with five highly experienced surgeons evaluating a VR application and considering it helpful, based on the System Usability Scale, with a score of 76.6% [34]. ML algorithms combined with VR have streamlined the annotation and quantification of medical images [35]. The average preoperative stomach volume was 794.93 mL, with volumes of 171.71 mL for postoperative laparoscopic sleeve gastrectomy and 35.73 mL for Roux-en-Y gastric bypass/one-anastomosis gastric bypass (RYGB/OAGB) gastric volumes for different surgical procedures [36].

AI for Imaging Segmentation

Deep learning approaches are highly accurate for the automated segmentation of anatomical structures for preoperative planning, thereby addressing the challenges of interpreting vast amounts of data generated by advanced imaging techniques [15]. AI algorithms, particularly deep learning models, have remarkable capabilities in automating anatomical structure segmentation, with the emergence of 3D CNN showing a significant evolution in deep learning applications and providing multidimensional analysis of medical images that surpasses traditional diagnostic methods [13,16].

In breast imaging applications, AI technologies enhance the detection capabilities and support clinical decision-making through efficient tumor size and volume measurements. Deep learning algorithms using the U-Net architecture help reduce clinician labor and variability in image analyses by advancing automatic segmentation [4]. Data from 48 cohort studies on breast imaging reveal impressive detection accuracy across various modalities, with mammography achieving an AUROC of 0.87, ultrasound demonstrating superior performance with an AUROC of 0.91, and MRI and digital breast tomosynthesis achieving comparable accuracy levels of 0.87 and 0.91, respectively [4]. CNNs have also been applied to improve detection rates in nuclear medicine imaging modalities for axillary lymph node detection and distant staging [4].

For nasal surgery applications, ML models, such as artificial neural networks, efficiently classify influencing factors and have been considered superior for detecting nasal bones because of their ability to rapidly depict the interdependence between nasal bones and facial landmarks. Various ML techniques, including backpropagation neural networks, random forests, and support vector machines, have been used to predict nasal problems and detect fractures by using CNNs and region-based CNNs [5].

Fully automated segmentation of the aortic valve surgery, using 3D transesophageal echocardiogram images, was achieved with significant time savings [37]. By using deep learning approaches for automatic segmentation, one study [37] reported an average Dice score of 0.95 for the aorta and left ventricle, 0.94 for the aorta and aortic valve, and 0.93 for the aortic root, aortic annulus, and sinotubular junction. Fully convolutional networks, which replace fully connected layers with convolutional layers and enable pixel-level segmentation, have been widely adopted for medical image segmentation [38].

Compared with manual approaches, which typically require 30-60 minutes, segmentation times with automated methods are significantly reduced, achieving segmentation in 12 seconds, 30 seconds, 45 seconds, and seven minutes across different studies [37]. A fully automated deep learning pipeline for thoracic aorta geometric analysis achieved automatic segmentation with a Dice score of 0.95 [37]. Trained U-Net models, such as TotalSegmentator and TotalSegmentator MRI (both: University of Freiburg, Freiburg, Germany), have been used to segment multiple anatomic structures in CT and MR images, respectively [38].

In specialized applications, 3D CNNs offer objectivity and standardization of diagnostic processes for ankle fracture detection, address variability in radiologist interpretations, and mitigate diagnostic error risks [16]. CNN demonstrated effectiveness in brain tumor segmentation, with a two-dimensional two-pathway cascading deep neural network achieving a state-of-the-art Dice score of 0.85 for glioblastoma segmentation in multi-modality MRIs [39]. A multi-label 3D U-Net achieved Dice scores exceeding 0.85 for subcortical volume segmentation from 3D fetal ultrasound [39].

Spiking neural networks have been successfully used in medical signal processing to encode spatiotemporal information with spike patterns, based on a leaky integrate-and-fire neuron model [40]. ML models for hepatocellular carcinoma achieved AUROC values of 0.8014 for pathological grade prediction and 0.980 (training) and 0.906 (validation) for microvascular invasion prediction, using 3D CNN approaches [41]. These advances in imaging segmentation support real-time intraoperative applications in which AI can monitor operations, identify anatomical structures, and help surgeons perform precise operations while reducing errors [7].

Discussion

Clinical Implications and Transformative Potential

The exceptional effectiveness of AI-powered risk classification systems demonstrated in this review, with AUROC scores surpassing 0.95 for predicting mortality and exceeding 0.80 for forecasting complications, signifies a transformative advancement in surgical risk evaluation. Reported AUROC values >0.90-0.95 for mortality and complication prediction suggest possible advantages over conventional tools in identifying at-risk patients. Similarly, high Dice scores and reduced segmentation times indicate that automated approaches may provide reliable and efficient preoperative imaging support [25]. These outcomes substantially exceed the performance of conventional assessment methodologies, aligning with cardiovascular surgery research in which ML algorithms markedly outperform EuroSCORE II and traditional logistic regression approaches [21]. Random forest algorithms have a performance of 84.7% precision and an AUROC of 87.3% [27], reflecting consistency with contemporary meta-analytical studies reporting comparable effectiveness measures across diverse surgical disciplines.

In reconstructive breast surgery, the integration of AI for DIEP flap planning has shown practical clinical utility, with semi-automatic algorithms reducing preoperative analysis time by two hours per patient while maintaining diagnostic accuracy [4]. This efficiency gain aligns with the broader literature showing the capacity of AI to optimize surgical workflows [4]. Gradient boosting algorithms have similarly demonstrated superior performance in predicting acute kidney injury with real-time risk assessment and in achieving high accuracy in perioperative risk stratification [24]. These findings exemplify the potential of ML-based predictive models to transform perioperative care delivery across surgical subspecialties through the earlier identification of at-risk patients and the optimization of resource allocation.

The emergence of AI-enhanced 3D simulation technologies has helped address longstanding challenges in surgical visualization and patient communication. Compared with patients who underwent traditional surgery, patients who underwent AI-assisted surgery experienced reduced operative times, decreased intraoperative blood loss, and fewer postoperative complications in one study [31]. These outcomes reflect findings from the orthopedic literature in which AI applications in total hip arthroplasty improved surgical precision and patient outcomes [13,14]. In rhinoplasty, ML algorithms trained on perioperative photographs offer more realistic outcome predictions than conventional simulation methods [5], thereby addressing the challenge of high revision rates owing to unmet patient expectations. The consistency of these benefits across surgical specialties suggests that AI-enhanced visualization represents a broadly applicable innovation in surgical planning.

Automated imaging segmentation may represent the most immediately applicable AI innovation, with this analysis showing a segmentation time reduction from 30-60 minutes to seconds while achieving Dice scores exceeding 0.95 for critical anatomical structures [37]. These performance metrics align with recent literature findings demonstrating consistent accuracy improvements across imaging modalities [37]. The AUROC of 0.91 achieved in breast ultrasound imaging and comparable performance across mammography, MRI, and digital breast tomosynthesis validate findings from larger meta-analyses showing the diagnostic superiority of AI over conventional approaches [4]. The combination of speed and accuracy improvements positions automated segmentation as a practical solution for reducing radiologist workload while maintaining or improving diagnostic quality.

However, most high-performance results come from retrospective, single-center studies, predominantly in cardiac, thoracic, and orthopedic surgery. Prospective multicenter validation remains limited.

Comparison With the Broader Literature

Our findings complement recent literature reports demonstrating the expanding role of AI across surgical specialties [23]. The 51% prevalence of risk prediction models among AI applications emphasizes predictive analytics as the most significant AI application in surgery, with 39% focusing specifically on risk assessment improvements [23]. However, this analysis demonstrated considerable progress in the integration of multimodal AI approaches, specifically shown through the synthesis of radiological image interpretation with clinical information to support holistic surgical planning. Thus, this development transcends stand-alone AI implementation toward comprehensive clinical decision-support frameworks.

The performance metrics observed in this review confirm the superiority of deep learning approaches. The emergence of specialized architectures, such as U-Net for medical image segmentation [38], has been validated across multiple surgical specialties, and these findings support their widespread adoption. AI-enhanced approaches have consistently demonstrated outcomes superior to those of traditional surgical planning methods. The patient tracking list tool's AUROC of 0.95 for mortality prediction [25] exceeded that of conventional risk stratification tools by substantial margins, advocating for AI-driven preoperative assessment protocols [25].

Fundamental Barriers and Implementation Challenges

Despite remarkable technological progress, multiple fundamental barriers have prevented the successful implementation of ML systems in clinical surgical practice. Research efforts predominantly consist of isolated institutional studies lacking comprehensive external validation, with <40% adhering to algorithm-specific reporting standards such as TRIPOD-AI [11]. This validation deficit represents a critical gap between promising research findings and clinical deployment, highlighting the translational research gap in AI applications in surgery [11].

Data integrity remains paramount because AI learning quality depends directly on data input precision, making the detection of fraudulent publications and data anomalies a critical concern [21]. The complex nature of AI systems makes interpreting their behavior challenging for surgical applications [21], thereby necessitating careful integration approaches that ensure that AI insights complement surgeons' expertise rather than overriding clinical judgment. The current verification mechanisms have significant limitations that directly undermine the data integrity required by AI systems for reliable learning [21].

Ethical barriers present significant challenges, with privacy, trust, consent, and conflicts of interest being primary concerns [22]. Confidentiality issues emerge when ML systems require extensive datasets for development, leading to conflicts with patient privacy protections. The General Data Protection Regulation of the European Union requires clear patient authorization for data utilization, highlighting the importance of transparent data management protocols [22]. These regulatory challenges align with the recent literature on bioethics, emphasizing the need for comprehensive governance frameworks in AI healthcare applications.

Professional confidence in ML systems is an additional obstacle because healthcare providers frequently lack adequate training to assess algorithmic software that often has insufficient clinical validation and transparency [22]. Algorithmic bias across diverse demographics remains insufficiently addressed [11], whereas the opacity of ML decision-making processes hinders the development of professional trust [22]. Recent studies [15] emphasize that robotic process automation protocols can overlook human emotional dynamics that are essential for effective physician-patient relationships.

Future Directions and Emerging Opportunities

The integration of AI with emerging technologies has provided exciting opportunities for advancement. AR-based and VR applications enhance surgical training and intraoperative guidance, with VR applications achieving a system usability scale score of 76.6% among experienced surgeons [34]. AI combined with 3D printing technologies has previously automated manual processes [32], making personalized surgical models more accessible and cost-effective, which is consistent with recent literature on additive manufacturing in surgery.

Future research should prioritize external validation studies across diverse patient populations and healthcare settings. Standardized reporting guidelines, particularly TRIPOD-AI adherence [11], are mandatory for AI research publications. The development of explainable AI frameworks offering clear reasoning pathways for clinical decisions is essential for widespread medical adoption, thereby resolving existing challenges in computational transparency [22]. Establishing robust ethical guidelines that address computational bias, information management protocols, and patient authorization is a critical imperative [22]. Incorporating AI education into surgical training programs will be vital for building professional expertise in AI-supported surgical practice, bridging current knowledge deficiencies among medical practitioners [22].

Limitations

The first limitation of this review was the heterogeneity of the studies reviewed, with many articles spanning various surgical disciplines such as general and cardiac surgery. The diversity of methodologies and AI applications reduces the ability to draw uniform conclusions specific to preoperative planning for surgery. Additionally, several studies lacked specific quantifiable data on preoperative surgery, and many of them did not include comprehensive datasets or long-term clinical data, which are crucial for a systematic review, but still represent a notable limitation in the context of a narrative review.

The second limitation is the lack of practical, real-world studies that apply AI in clinical settings. Although numerous investigations have established the conceptual capabilities of AI systems, a considerable research gap exists regarding their practical deployment in standard surgical procedures. Additionally, the accelerated evolution of AI technologies indicates that certain findings may rapidly lose relevance, thereby constraining the sustained applicability of specific research outcomes. These considerations underscore the necessity for future multicenter investigations employing standardized assessment criteria, real-world deployment research, and comprehensive validation methodologies to address these constraints and deliver more conclusive evidence regarding the function of AI in surgical practice.

## Conclusions

This narrative review examined the reported potential efficacy and constraints of AI in preparation for surgical procedures. AI methodologies, which include ML algorithms, 3D modeling, and predictive analytics, have demonstrated considerable potential for enhancing surgical accuracy, thereby minimizing postoperative complications and improving patient outcomes across diverse plastic surgery interventions. However, obstacles persist, such as ethical considerations, dataset biases, and deployment challenges, which impede broad clinical acceptance. Despite these barriers, AI applications can transform surgical preparation by supporting clinical decision-making processes and optimizing the procedural workflow. Additionally, various emerging technologies were identified, such as 3D printing and VR systems; these technologies may advance the application of AI in surgical practice. Subsequent research should prioritize addressing the identified constraints, particularly regarding practical implementation in clinical settings, and investigate the integration of AI with innovative technologies. Resolving these challenges and validating AI frameworks via comprehensive multicenter investigations will prove crucial for achieving the complete potential of AI in revolutionizing plastic surgery interventions.
